# Post Pharyngeal Abscess

**Published:** 1892-05-14

**Authors:** 


### POST-PHARYNGEAL ABSCESS.

Post-pharyngeal abscess is one of those conditions which greater accuracy in diagnosis proves to be considerably more common than the meagre description usually found in the various surgical text books warrants us in supposing.

Infants and very young children are those who most commonly suffer, and there is little doubt but that this complaint is very frequently put down as croup, spinal meningitis, marasmus or convulsions, for the symptoms are often very equivocal unless the physical signs are more carefully elicited than is usually the case with restless crying babies.

If we pin our faith upon the text books, we shall believe the cause almost always dependent upon spinal caries; practical experience, however, shows that, on the contrary, this is an extremely rare cause. In four clear cases which came under the writer's notice within three months of the past winter, there were no signs whatever of spinal disease, and the patients became perfectly well after treatment. Out of 204 cases observed at the Children's Hospital in Pesth, only 7 were dependent upon spinal caries. Of course, there is no doubt that tubercular disease of the atlas, or axis, or cervical vertebræ will cause post-pharyngeal abscess, but these are situated more deeply beneath the ligaments and fascia in close contact with the vertebræ, and not in the connective tissue between the pharynx and the fascia covering the pre-vertebral muscles, which is the site of the usual variety.

Treves has pointed out that in this lax connective tissue and opposite the axis, a lymphatic gland may be found which receives lymphatics from the nares.

Simon observes also that the lymphatics of the post-pharyngeal region form networks on each side, which terminate in glands located one on each side of the median line between the pharynx and prevertebral aponeurosis. He further states that these glands disappear altogether after the third year of life, which is in striking agreement with the clinical experience that the majority of children attacked ara quite young.

The anatomy being thus understood the cause is easily explained. Nasal catarrhs are exceedingly frequent in young children. A simple adenitis is set up, which may terminate in suppuration, with the result that the pus pushes the posterior wall of the pharynx forwards, depressing the soft palate, causing a swelling in the throat, and also, as is usually the case, by travelling along the planes of the cervical fascia, and passing under the parotid gland and vessels of the neck, it points beneath, or at either border of the sterno-mastoid muscle. Acute phlegmonous inflammation may also induce the same condition. In one of the writer's cases, a boy of five years old, who had been suffering for six months great inconvenience from the disease, the symptoms dated from recovery after scarlet fever. It is possible, indeed highly probable, that such inflammation may be caused in the pharynx by some slight scratch from a foreign body during deglutition, or from injury caused by the invariable but reprehensible habit babies have of pushing long sticks, ornaments, or anything they can lay their hands upon, into their mouths. Any septic material present might easily be absorbed, if the integrity of the pharyngeal mucous membrane be impaired by even the slightest abrasion.

With a little tact and judgment there is no difficulty in recognising the disease. The chief points to be noted are as follows: The onset is often insidious, and passes for some time unnoticed, frequently it simulates an attack of tonsillitis; there is generally much dyspnœa, which is sometimes very intense, with lividity of face, and marked recession of chest. Respiration is noisy and snoring, especially during sleep, and the child awakes now and then with a sudden start. A brassy croup-like cough may be present, with much yellowish, frothy expectoration. Dysphagia is always a notable symptom; the child, though eager for its food, has the greatest possible difficulty in swallowing it, all attempts in severe cases being accompanied by fits of choking and regurgitation, sometimes food returning through the nose.

Phonation may be indistinct, the voice sounding hoarse and thick from intercurrent laryngitis.

Usually there is marked retraction of head, and a swelling is generally present in the neck underneath or at one border of the sterno-mastoid, which is sometimes mistaken for enlarged glands. A history of much loss of flesh is nearly always elicited.

On examination of the mouth the tongue is seen to be coated with a white fur, and there is usually much frothy yellowish mucus, which prevents a good view of the pharynx being obtained. But on gentle palpation with the forefinger, a distinct tense elastic swelling is felt—unilaterally in most cases—beyond the tonsils, at the back of the pharynx, and occasionally fluctuation may be discovered between this swelling and the one in the neck.

When a child is found suffering from the above cardinal symptoms of dyspnœa, dysphagia, retraction of head, with a swelling in the neck, the probability is we are dealing with a case of pharyngeal abscess, and if a swelling is also seen or felt in the pharynx, the diagnosis is complete.

The treatment from the first moment pus appears is mainly surgical. Should a case be diagnosed in the early stage before suppuration, it should be kept under observation to await events. If the child is old enough warm gargles are useful, and the swelling may be painted with weak iodine solution. Saline aperients are indicated with plenty of good nourishing food.

Directly pus is diagnosed, surgical interference should be adopted without delay, for the swelling may increase rapidly, giving rise to the serious symptoms above enumerated; and it may also, by bursting, especially during sleep, or when help is not at hand, cause death from suffocation, the matter being sucked into the larynx.

Usually the operation is very simple and consists in opening the abscess either in the neck, or as most of the older surgeons taught, by the mouth. If opened in the latter situation the child should be put under chloroform, its mouth kept open by a gag, and the head allowed to hang down over the end of the operating table. The abscess may then be aspirated, or preferably, a longitudinal incision made along the whole length of the swelling, the pus being rapidly sponged away to prevent it flowing into the larynx.

There are several objections to this method, the abscess cavity cannot be drained through the mouth, consequently the wound tends to heal and the pus to re-accumulate; proper antiseptic treatment is, of course, impossible, and the discharge is being continually swallowed. Consequently there can be no doubt that opening the abscess from the neck is by far the best plan, and in the hands of a careful surgeon there is no serious difficulty in the operation.

Hilton, as far back as 1860, advanced his own method of opening post-pharyngeal abscess. He says, "I carefully made an incision, about half an inch in length, with a lancet, through the sterno-cleido-mastoid, thus exposing the fascia underneath it; I then thrust a grooved probe or director through the fascia towards the back part of the pharynx, when a little stream of opaque fluid came trickling down the director. I then ran the dressing forceps along the grooved director, made an opening into the deep abscess, and let out three or four ounces of pus. The exit of pus was aided by passing my finger into the child's mouth, and pressing upon the posterior wall of her pharynx."

Even in young infants, and where the abscess is small and confined to the post-pharyngeal tissue, this procedure may be used with equally good results. The incision is best made below the mastoid process along the posterior border of the sterno-mastoid muscle, a cautious dissection with blunt instruments should be made, guided by one finger placed in the mouth on the pharynx. A director is then thrust into the abscess cavity, and the opening enlarged in the usual way by sinus forceps, a drainage tube inserted, and the wound treated antiseptically. As a rule, the results are all that can be desired, the distressing symptoms being immediately relieved, and the patient regains strength rapidly.

